# Racial and Ethnic Disparities in the Management of Postdural Puncture Headache With Epidural Blood Patch for Obstetric Patients in New York State

**DOI:** 10.1001/jamanetworkopen.2022.8520

**Published:** 2022-04-21

**Authors:** Allison Lee, Jean Guglielminotti, Anne-Sophie Janvier, Guoha Li, Ruth Landau

**Affiliations:** 1 Department of Anesthesiology, Columbia University Irving Medical Center, New York, New York; 2 Department of Epidemiology, Columbia University Mailman School of Public Health, New York, New York

## Abstract

**Importance:**

Characterizing and addressing racial and ethnic disparities in peripartum pain assessment and treatment is a national priority.

**Objective:**

To evaluate the association of race and ethnicity with the provision and timing of an epidural blood patch (EBP) for management of postdural puncture headache in obstetric patients.

**Design, Setting, and Participants:**

This cross-sectional study used New York State hospital discharge records from January 1, 1998, to December 31, 2016, from mothers 15 to 49 years of age with a postdural puncture headache after neuraxial analgesia or anesthesia for childbirth. Statistical analysis was performed from February 2020 to February 2022.

**Exposures:**

Patients’ race and ethnicity (reported as provided by each participating hospital; the method of determining race and ethnicity [ie, self-reported or not] cannot be determined from the data) were categorized into non-Hispanic White (reference group), non-Hispanic Black, Hispanic, and other race and ethnicity (including Asian and Pacific Islander, American Indian, Alaskan Native, and other).

**Main Outcomes and Measures:**

The primary outcome was the rate of EBP use. The secondary outcome was the interval (days) between hospital admission and provision of EBP. Odds ratios (ORs) and 95% CIs of EBP use associated with race and ethnicity were estimated using mixed-effect logistic regression models, adjusting for patient and hospital characteristics.

**Results:**

During the study period, 8921 patients (mean [SD] age, 30 [6] years; 1028 [11.5%] Black; 1301 [14.6%] Hispanic; 4960 [55.6%] White; and 1359 [15.2%] other race and ethnicity) with postdural puncture headache were identified among 1.9 million deliveries with a neuraxial procedure. Of these 8921 patients, 4196 (47.0%; 95% CI, 46.0%-48.1%) were managed with an EBP. A total of 2650 White patients (53.4%; 95% CI, 52.0%-54.8%) used an EBP; this rate was significantly higher than that among Hispanic patients (41.7% [543]; 95% CI, 39.9%-44.5%), Black patients (35.7% [367]; 95% CI, 32.8%-38.7%), or patients of other race and ethnicity (35.2% [478]; 95% CI, 32.6%-37.8%). Timing of EBP was at a median of 2 days (IQR, 2-3 days) after hospital admission for White patients compared with a median of 3 days (IQR, 2-4 days) for Hispanic patients, Black patients, and patients of other race and ethnicity (*P* < .001 for the comparison with White patients). After adjustment for patient and hospital characteristics, the EBP rate was not different between White and Hispanic patients (adjusted OR, 1.11; 95% CI, 0.94-1.30). It was significantly lower for Black patients (adjusted OR, 0.80; 95% CI, 0.67-0.94) and patients of other races and ethnicities (adjusted OR, 0.85; 95% CI, 0.73-0.99).

**Conclusions and Relevance:**

In this study, significant racial and ethnic disparities in the management of postdural puncture headache with EBP were observed, with both lower rates and delayed timing, which may be associated with long-term adverse effects.

## Key Points

**Question:**

Do patients in racial and ethnic minority groups giving birth receive an epidural blood patch for management of postdural puncture headache after neuraxial procedures less frequently than non-Hispanic White patients giving birth?

**Findings:**

This cross-sectional study found that, among the 8921 cases of obstetric postdural puncture headache identified in New York State hospitals, an epidural blood patch was used less often or its use was delayed for patients in racial and ethnic minority groups compared with non-Hispanic White patients.

**Meaning:**

These findings suggest that there is a need to address racial and ethnic disparities in the management and prevention of serious complications associated with obstetric anesthesia care.

## Teaser

This cross-sectional study evaluates the association of race and ethnicity with the provision and timing of an epidural blood patch for management of postdural puncture headache in obstetric patients.

## Introduction

Addressing racial and ethnic disparities in pain management during childbirth and during the postpartum period is a priority in the US.^1^ Compared with non-Hispanic White patients, patients in racial and ethnic minority groups have a 60% increased risk of experiencing severe postpartum pain and are 20% less likely to receive opioids for postpartum pain.^2,3^ Compared with patients with mild postpartum pain, those with severe pain have a 3.0-fold increased risk of depression at 8 weeks post partum.^4^ Clinicians’ implicit bias toward obstetric patients in racial and ethnic minority groups is speculated to account for these disparities.^5,6^

Postdural puncture headache (PDPH) is the most common complication of neuraxial procedures (ie, spinal, epidural, or combined spinal epidural techniques) performed for childbirth, affecting up to 1.5% of patients who underwent these procedures.^7,8^ Postdural puncture headache is characterized by severe positional headache that may slow maternal recovery, delay hospital discharge, and limit the ability of mothers to care for and bond with their newborns and that may lead to chronic headaches,^9^ postpartum depression, and central nervous system complications.^4,10,11^ With 3.5 million annual births in the US and approximately 80% of pregnant people receiving neuraxial labor analgesia or anesthesia, PDPH and its complications may be associated with a significant disease burden. The epidural blood patch (EBP) is the criterion standard treatment for severe PDPH.^12^ Despite some benefit to trialing conservative therapy, evidence indicates that an EBP procedure should be performed early and repeated if indicated.^13^ In view of reported racial and ethnic disparities in the evaluation and treatment of peripartum pain, we hypothesized that EBP rates for the management of PDPH would be lower and delayed for patients in racial and ethnic minority groups compared with non-Hispanic White patients.

## Methods

The study protocol was granted exemption under 45 Code of Federal Regulation 46 (not human participants research) by the Columbia University Irving Medical Center institutional review board because the data are deidentified. Because the data are deidentified, it is not possible to obtain informed consent. The Strengthening the Reporting of Observational Studies in Epidemiology (STROBE) reporting guideline was followed, and the Reporting of Studies Conducted Using Observational Routinely-Collected Health Data (RECORD) statement was used.

### Data Systems

Data for this study came from the State Inpatient Database (SID) for New York. State inpatient databases are part of the Healthcare Cost and Utilization Project (HCUP) sponsored by the Agency for Healthcare Research and Quality.^14^ They capture all inpatient discharges from nonfederal acute care community hospitals, including tertiary and academic centers. For each discharge, HCUP SID data provide some patient characteristics (eg, race and ethnicity), the American Hospital Association (AHA) hospital identifier, and patient diagnoses and procedures defined in the *International Classification of Diseases, Ninth Revision, Clinical Modification* (*ICD-9-CM*) and the *International Statistical Classification of Diseases and Related Health Problems, Tenth Revision, Clinical Modification* (*ICD-10-CM*). The *ICD-10-CM* was implemented in October 2015. Furthermore, New York is the only state among the 33 HCUP SID participating states to provide information on anesthesia care. Anesthesia care is reported as a categorical variable with values corresponding to no anesthesia care, local anesthesia, general anesthesia, regional anesthesia (ie, neuraxial), other anesthesia, and missing. Each discharge record contains a maximum of 1 value for anesthesia care.

Hospital characteristics were calculated using HCUP SID data (eg, annual volume of deliveries) or directly abstracted from the AHA Annual Survey Database (eg, teaching hospital). The AHA survey is a census of US hospitals, providing comprehensive information on hospital characteristics.^15^ Information on health care professionals (eg, number of physician anesthesiologists) at the hospital and county level was abstracted from the Area Health Resource File provided by the US Health Resources and Services Administration.^16^

### Study Sample

The study sample included delivery hospitalizations among pregnant patients 15 to 49 years of age who received neuraxial analgesia or neuraxial anesthesia during childbirth and who experienced PDPH between January 1, 1998, and December 31, 2016. Delivery hospitalizations were identified with a combination of *ICD-9-CM* and *ICD-10-CM* algorithms.^17^ Postdural puncture headache was identified using the *ICD-9-CM* diagnosis code 349.0 (“headache following lumbar puncture”) and *ICD-10-CM* diagnosis codes O74.5 (“spinal and epidural anesthesia–induced headache during labor and delivery”), O89.4 (“spinal and epidural anesthesia–induced headache during the puerperium”), and G97.1 (“other reaction to spinal and lumbar puncture [headache]”).^10,11^

### Exposures

The exposure of interest was patients’ race and ethnicity. In HCUP SID data, race and ethnicity are categorized into 7 mutually exclusive groups: (1) non-Hispanic White (hereinafter referred to as White), (2) non-Hispanic Black (Black or African American), (3) Hispanic, (4) non-Hispanic Asian or Pacific Islander (Asian or Pacific Islander), (5) non-Hispanic Native American (Native American), (6) other race and ethnicity, and (7) missing. A patient’s race and ethnicity are reported as provided by each participating hospital, and data do not indicate whether race and ethnicity were self-reported or not by the patient. Because of low counts, Asian or Pacific Islander patients, Native American patients, and patients of other race and ethnicity were combined into a unique category (other race and ethnicity). Patients with missing information on race and ethnicity were not excluded but analyzed as an independent racial and ethnic group (missing race and ethnicity).

### Outcomes

The primary outcome was the EBP rate, identified using the *ICD-9-CM* procedure code 03.95 (“spinal blood patch”) and the *ICD-10-CM* procedure code 3E0S3GC (“introduction of other therapeutic substance into epidural space, percutaneous approach”).^10,11^ The secondary outcome was the interval between hospital admission and EBP. The HCUP SID data provide a variable (*PRDAYn* or number of days from admission to procedure *n*) that indicates the number of days elapsed between hospital admission and the procedure *n*, but not the interval between a neuraxial procedure and the EBP. We assumed that the admission day corresponded to the day of the neuraxial procedure for labor analgesia or operative procedure.

### Maternal and Hospital Characteristics

The following maternal characteristics were recorded directly from the HCUP SID data: age, health insurance type (categorized as Medicaid or Medicare, private, self-pay [uninsured], and other), and admission during a weekend. Individual comorbid conditions were summarized using the comorbidity index for obstetric patients calculated using previously described *ICD-9-CM* and *ICD-10-CM* algorithms.^18,19^ This index includes maternal age and 20 maternal conditions (eg, severe preeclampsia) that are associated with maternal end-organ injury or death during the delivery hospitalization through 30 days post partum. Maternal obesity, cesarean delivery, and possible contraindications to neuraxial procedures were identified using *ICD-9-CM* and *ICD-10-CM* codes (eTable 1 in the Supplement).

For each hospital, the following characteristics were calculated for each year of the study period in all deliveries using HCUP SID data: volume of delivery, cesarean delivery rate, proportion of obstetric patients in racial and ethnic minority groups, proportion of safety-net patients (including proportion of Medicaid beneficiaries, Medicare beneficiaries, and uninsured patients), proportion of patients admitted for delivery during a weekend, proportion of neuraxial procedures for childbirth, and coding intensity. For each hospital, coding intensity was calculated as the mean number of diagnoses and procedure codes reported per discharge.^20^ Adjustment based on coding intensity accounts for the marked variations in coding patterns across hospitals and over time.^21,22^ Hospital location (rural or urban) and teaching status were abstracted from the AHA Annual Survey Database. Rural location included micropolitan or noncore areas based on the Core-Based Statistical Areas. A teaching hospital had an affiliation to a medical school or residency training accreditation. The numbers of obstetricians, gynecologists, and physician anesthesiologists per 1000 in-hospital births were abstracted at the hospital county level from the Area Health Resource File for each year of the study period.

### Statistical Analysis

Statistical analysis was performed from February 2020 to February 2022 with R, version 4.0.3 (R Group for Statistical Computing) and packages lme4 for mixed-effect models and mice for multiple imputations. Statistical tests were 2-sided tests. No a priori power calculation was performed.

#### Univariate Analysis

Results are reported as mean (SD), median and IQR, or count and percentage. Patients giving birth with EBP and those without EBP were compared using the χ^2^ test for categorical variables, the Wilcoxon rank sum test for continuous variables, and the standardized mean difference. A standardized mean difference greater than 10% was used to define a statistically significant imbalance.^23^ Comparisons of the interval between hospital admission and EBP across the 5 racial and ethnic groups (Black, Hispanic, White, other, and missing) were performed using the Kruskal-Wallis rank sum test with a significance threshold at *P* = .05. Comparisons of the interval between hospital admission and EBP between patients in each racial and ethnic minority group (Black, Hispanic, other, and missing) and the White patients were performed using the Wilcoxon rank sum test with a significance threshold at *P* = .0125 (.05/4). Crude odds ratios (ORs) of EBP use associated with race and ethnicity were estimated using mixed-effect logistic regression models, with EBP use as the dependent variable, race and ethnicity as the independent variable, and the hospital identifier as the random effect (random intercept and constant slope).

#### Multivariate Analysis

Odds ratios of EBP use were adjusted for the 20 patient- and hospital-level characteristics reported in Table 1, along with the year of delivery. Missing values for patient- and hospital-level characteristics were handled using multiple imputations (eTable 2 in the Supplement).

**Table 1.** Comparison of Mothers With or Without EBP for PDPH After Neuraxial Analgesia or Anesthesia for Childbirth (New York State Hospitals, 1998-2016)

Characteristic	Missing, No.	No. (%)		*P* value	Absolute SMD, %
		PDPH without EBP (n = 4725)	PDPH with EBP (n = 4196)		
Patient characteristics					
Age, mean (SD), y	0	30 (6)	30 (6)	.74	<0.1
Health insurance					
Medicaid or Medicare	0	1948 (41.2)	1254 (29.9)	<.001	28.0
Private	0	2548 (53.9)	2822 (67.3)		
Self-pay (uninsured)	0	128 (2.7)	55 (1.3)		
Other	0	101 (2.1)	65 (1.5)		
Obesity	0	258 (5.5)	169 (4.0)	.002	6.7
Comorbidity index for obstetric patients, mean (SD)	0	1.6 (2.7)	1.4 (2.6)	<.001	8.3
Delivery					
Admission for delivery during a weekend	0	799 (16.9)	790 (18.8)	.02	5.0
Cesarean delivery	0	3217 (68.1)	2427 (57.8)	<.001	21.3
Contraindications to neuraxial techniques					
Coagulation factor deficit, Von Willebrand disease, and thrombocytopenia	0	70 (1.5)	49 (1.2)	.23	2.7
Fever or infection during labor	0	36 (0.8)	47 (1.1)	.01	3.7
Chorioamnionitis	0	122 (2.6)	79 (1.9)	.03	4.7
Hospital characteristics					
Teaching hospital, No./total No. (%)	1153	3115/4109 (75.8)	2516/3659 (68.8)	<.001	15.8
Rural hospital, No./total No. (%)	1153	201/4109 (4.9)	268/3659 (7.3)	<.001	10.2
Volume of delivery, mean (SD)	88	2724 (1721)	2703 (1769)	.57	1.2
Cesarean delivery rate, mean (SD)	88	32.2 (7.4)	32.2 (7.8)	.64	1.0
Proportion of racial and ethnic minority parturients, mean (SD)	178	51.5 (32.0)	38.4 (27.1)	<.001	44.1
Proportion of safety-net parturients, mean (SD)^a^	88	46.5 (27.7)	36.8 (22.2)	<.001	38.5
Proportion of admissions for delivery during a weekend, mean (SD)	88	20.7 (2.4)	20.4 (2.4)	<.001	11.6
Proportion of neuraxial analgesia or anesthesia for delivery, mean (SD)	88	45.9 (32.8)	52.6 (31.6)	<.001	20.8
Coding intensity in deliveries, mean (SD)	88	7.4 (1.8)	7.2 (1.7)	<.001	9.6
Hospital county characteristics (per 1000 in-hospital births in the county)					
No. of obstetricians and gynecologists, mean (SD)	1510	14.1 (5.0)	13.7 (4.9)	.001	7.5
No. of physician anesthesiologists, mean (SD)	1510	15.2 (6.5)	15.3 (6.4)	.73	<0.1

Abbreviations: EBP, epidural blood patch; PDPH, postdural puncture headache; SMD, standardized mean difference.

^a^ Proportion of Medicaid beneficiaries, Medicare beneficiaries, and uninsured in all deliveries.

To assess the robustness of our findings, we conducted 4 sensitivity analyses: (1) handling of missing values for patient- and hospital-level characteristics using a complete case analysis instead of multiple imputations, (2) exclusion of hospitals with a proportion of missing values for maternal race and ethnicity greater than 20%, (3) exclusion of hospitals with a proportion of missing values for the number of obstetricians and gynecologists greater than 20%, and (4) exclusion of hospital discharges in 2016 because of the introduction of the *ICD-10-CM* classification in October 2015. Hospitals with a proportion of missing values greater than 20% were identified among all delivery hospitalizations and not in the study sample.

## Results

During the study period, 8921 patients (mean [SD] age, 30 [6] years; 1028 [11.5%] Black; 1301 [14.6%] Hispanic; 4960 [55.6%] White; and 1359 [15.2%] other race and ethnicity) with PDPH were identified (eFigure in the Supplement). Of these 8921 patients, 4196 (47.0%; 95% CI, 46.0%-48.1%) received an EBP. Compared with patients with PDPH who did not receive an EBP (n = 4725), those who received an EBP were more likely to have private health insurance and undergo a vaginal delivery (Table 1). They were also more likely to have given birth in a nonteaching hospital, a rural hospital, a hospital with a lower proportion of patients in racial and ethnic minority groups, a hospital with a lower proportion of safety-net patients, and a hospital with a higher rate of neuraxial procedures during childbirth.

A total of 2650 White patients with PDPH (53.4%; 95% CI, 52.0%-54.8%) used an EBP compared with 543 Hispanic patients (41.7%; 95% CI, 39.9%-44.5%), 367 Black patients (35.7%; 95% CI, 32.8%-38.7%), and 478 of 1359 patients of other race and ethnicity (35.2%; 95% CI, 32.6%-37.8%) (Figure and Table 2). Consequently, the EBP rate was 8% lower for Hispanic patients giving birth (unadjusted OR, 0.92; 95% CI, 0.80-1.07), 32% lower for Black patients giving birth (unadjusted OR, 0.68; 95% CI, 0.58-0.80), and 28% lower for patients of other race and ethnicity giving birth (unadjusted OR, 0.72; 95% CI, 0.62-0.83) (Table 2).

**Figure.**
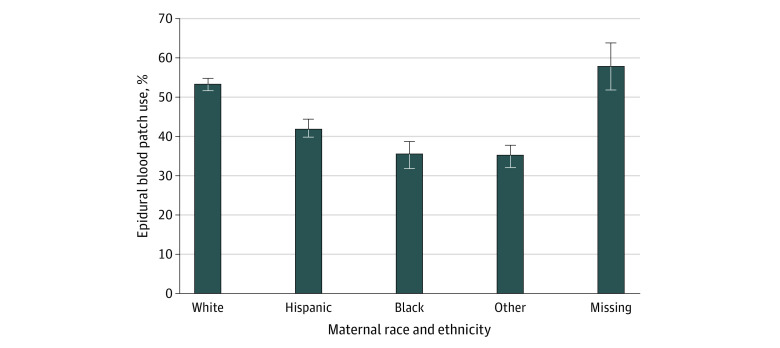
Use of Epidural Blood Patch for Postdural Puncture Headache After Neuraxial Analgesia or Anesthesia for Childbirth According to Maternal Race and Ethnicity (New York State Hospitals, 1998-2016) Other includes Asian and Pacific Islander, Native American, and other race and ethnicity. Error bars indicate 95% CIs.

**Table 2.** Crude and Adjusted Odds Ratios of EBP Use for PDPH After Neuraxial Analgesia or Anesthesia for Childbirth (New York State Hospitals, 1998-2016)

Race and ethnicity	PDPH without EBP (n = 4725)		PDPH with EBP (n = 4196)		*P* value	Odds ratio (95% CI)	
	Count	% (95% CI)	Count	% (95% CI)		Crude^a^	Adjusted^b^
White	2310	46.6 (45.2-48.0)	2650	53.4 (52.0-54.8)	<.001	1 [Reference]	1 [Reference]
Hispanic	758	58.3 (55.5-61.0)	543	41.7 (39.9-44.5)		0.92 (0.80-1.07)	1.11 (0.94-1.30)
Black	661	64.3 (61.3-67.2)	367	35.7 (32.8-38.7)		0.68 (0.58-0.80)	0.80 (0.67-0.94)
Other^c^	881	64.8 (62.2-6.4)	478	35.2 (32.6-37.8)		0.72 (0.62-0.83)	0.85 (0.73-0.99)
Missing	115	42.1 (36.2-48.2)	158	57.9 (51.8-63.8)		1.29 (0.98-1.70)	1.31 (0.98-1.75)

Abbreviations: EBP, epidural blood patch; PDPH, postdural puncture headache.

^a^ Estimated using univariate mixed-effect logistic regression with the hospital identifier as the random effect (random intercept and constant slope).

^b^ Adjusted for the following 21 characteristics: (1) maternal age; (2) health insurance; (3) obesity; (4) comorbidity index for obstetric patients; (5) admission during a weekend; (6) cesarean delivery; (7) coagulation factor deficit, Von Willebrand disease, and thrombocytopenia; (8) fever or infection during labor; (9) chorioamnionitis; (10) teaching hospital; (11) rural hospital; (12) hospital annual volume of delivery; (13) hospital cesarean delivery rate; (14) hospital proportion of racial and ethnic minority obstetric patients; (15) hospital proportion of safety-net parturients; (16) hospital proportion of admissions for delivery during a weekend; (17) hospital proportion of patients receiving neuraxial analgesia or anesthesia for delivery; (18) hospital coding intensity in deliveries; (19) hospital county number of obstetricians and gynecologists; (20) hospital county number of physician anesthesiologists; and (21) year of delivery. Missing values were handled using multiple imputations.

^c^ Includes Asian and Pacific Islander, Native American, and other race and ethnicity.

Use of an EBP occurred a median of 2 days (IQR, 2-3 days) after hospital admission for White patients compared with a median of 3 days (IQR, 2-4 days) after hospital admission for Hispanic patients, Black patients, and patients of other race and ethnicity, which was significantly different for the 3 minoritized groups compared with White patients (*P* < .001) (Table 3). After adjustment for patient and hospital characteristics, the EBP rate was not different between White and Hispanic patients (adjusted OR, 1.11; 95% CI, 0.94-1.30) (Table 2; eTable 3 in the Supplement) but was significantly lower for Black patients (adjusted OR, 0.80; 95% CI, 0.67-0.94) and for patients of other race and ethnicity (adjusted OR, 0.85; 95% CI, 0.73-0.99). Results were similar in the 4 sensitivity analyses (Table 4).

**Table 3.** Interval Between Hospital Admission and Epidural Blood Patch for Postdural Puncture Headache After Neuraxial Analgesia or Anesthesia for Childbirth (New York State Hospitals, 1998-2016)

Race and ethnicity	Interval, median (IQR), d^a^	*P* value	
		For comparison with White patients^b^	Overall^c^
Black	3 (2-4)	<.001	<.001
Hispanic	3 (2-4)	<.001	
White	2 (2-3)	NA	
Other^d^	3 (2-4)	<.001	
Missing	2 (2-3)	.59	

Abbreviation: NA, not applicable.

^a^ The information on the interval is missing for 54 of the 4196 epidural blood patches (1.3%).

^b^ Significance threshold at *P* = .0125 (.05/4).

^c^ Significance threshold at *P* = .05.

^d^ Includes Asian and Pacific Islander, Native American, and other race and ethnicity.

**Table 4.** Adjusted Odds Ratios of Epidural Blood Patch Use for Postdural Puncture Headache After Neuraxial Analgesia or Anesthesia for Childbirth in the Main and in the 4 Sensitivity Analyses (New York State Hospitals, 1998-2016)

Characteristic	Adjusted odds ratio (95% CI)^a^				
	Main analysis (imputation of missing values)	Sensitivity analysis 1 (complete case analysis)	Sensitivity analysis 2 (exclusion of hospitals with a proportion of missing values for maternal race and ethnicity >20%)	Sensitivity analysis 3 (exclusion of hospitals with a proportion of missing values for hospital number of obstetricians and gynecologists >20%)	Sensitivity analysis 4 (exclusion of discharges in 2016)
No. of observations	8921	7213	8647	8492	8486
No. of hospitals	158	149	154	137	158
Maternal race and ethnicity					
White	1 [Reference]	1 [Reference]	1 [Reference]	1 [Reference]	1 [Reference]
Hispanic	1.11 (0.94-1.30)	1.17 (0.98-1.40)	1.10 (0.94-1.30)	1.10 (0.93-1.30)	1.10 (0.93-1.30)
Black	0.80 (0.67-0.94)	0.81 (0.67-0.97)	0.80 (0.68-0.95)	0.81 (0.68-0.96)	0.79 (0.66-0.94)
Other^b^	0.85 (0.73-0.99)	0.84 (0.71-0.99)	0.84 (0.72-0.98)	0.85 (0.73-0.99)	0.85 (0.73-0.99)
Missing	1.31 (0.98-1.75)	1.73 (1.16-2.58)	1.52 (1.07-2.15)	1.31 (0.98-1.75)	1.26 (0.94-1.68)

^a^ Adjusted for the following 21 characteristics: (1) maternal age; (2) health insurance; (3) obesity; (4) comorbidity index for obstetric patients; (5) admission during a weekend; (6) cesarean delivery; (7) coagulation factor deficit, Von Willebrand disease, and thrombocytopenia; (8) fever or infection during labor; (9) chorioamnionitis; (10) teaching hospital; (11) rural hospital; (12) hospital annual volume of delivery; (13) hospital cesarean delivery rate; (14) hospital proportion of racial and ethnic minority obstetric patients; (15) hospital proportion of safety-net parturients; (16) hospital proportion of admissions for delivery during a weekend; (17) hospital proportion of patients receiving neuraxial analgesia or anesthesia for delivery; (18) hospital coding intensity in deliveries; (19) hospital county number of obstetricians and gynecologists; (20) hospital county number of physician anesthesiologists; and (21) year of delivery. Missing values were handled using multiple imputations.

^b^ Includes Asian and Pacific Islander, Native American, and other race and ethnicity.

## Discussion

To our knowledge, this is the first study to evaluate the association between patients’ race and ethnicity and the provision of EBP for PDPH among obstetric patients in the US and to report marked disparities by race and ethnicity. Rates of EBP for the management of PDPH in Black patients and patients in other racial and ethnic minority groups were significantly lower compared with White patients. When management of PDPH with EBP for patients in racial and ethnic minority groups did happen, EBP was performed a median of 1 day later compared with White patients.

Postdural puncture headache is a well-recognized complication of neuraxial procedures.^24^ The cause is presumed to be loss of cerebrospinal fluid through the dural hole, resulting in intracranial hypotension; traction on cranial structures, leading to compensatory cerebral vasodilation; and headache that can be severe and incapacitating.^25^ An EBP is expected to relieve pain through a tamponade effect, causing an increase in intracranial cerebrospinal fluid pressure and/or the promotion of sealing of the dural defect.^12,26^ Delay in treatment of PDPH is problematic because it not only results in pain for the patient, debilitating headache, and incapacity to care for the newborn but is associated with severe morbidity and significantly increased risk of chronic headaches,^9^ cerebral venous thrombosis, subdural hematoma, bacterial meningitis, postpartum depression, persistent headache, and persistent low-back pain.^10^

Our observational cross-sectional study design precludes determination of causal factors, but the factors associated with inequities may be conceptualized at the level of the patient, clinician, clinical encounter, and health care system.^27^ Patient preferences, refusal of treatment, clinician bias, language barriers, and quality of communication could not be assessed. Among Black patients, we can speculate about the possible contributions of medical mistrust^28^ as well as the perceptions of racial bias that are common among Black patients during health care interactions.^29^ We can also speculate that fewer pain assessments might be performed for patients in racial and ethnic minority groups giving birth, similar to prior reports regarding post–cesarean delivery pain evaluation and management,^3^ underscoring the need for postprocedural follow-up protocols so that patients with PDPH may be identified early and offered the option of receiving an EBP.

Women in racial and ethnic minority groups have lower rates of perinatal health insurance coverage^30^; however, lack of insurance is not a likely explanation of our findings. Pregnant individuals who are Black are more likely to give birth in lower-quality, higher-risk–standardized severe maternal morbidity hospitals than pregnant individuals who are White, the underlying reasons for which are complex but include long-standing patterns of racially segregated neighborhoods that have experienced chronic underinvestment.^31,32^ Reports of differential outcomes within hospital types suggest that differential care may be occurring^33^; the persistence of disparate outcomes after adjustment for socioeconomic status and health care access points to deeper structural and societal factors, including structural racism.^34^

Our findings of lower EBP rates and delays in providing EBP for patients in racial and ethnic minority groups extend our current knowledge regarding disparities in obstetric anesthesia care.^34,35,36,37^ Previous research has identified that patients in racial and ethnic minority groups are less likely to use neuraxial labor analgesia—50% of Black patients and 35% of Hispanic patients compared with 60% of White patients—despite the fact that neuraxial labor analgesia is the most effective and safe form of analgesia available for labor and delivery for both mothers and newborns.^38^ Similarly, although neuraxial anesthesia is considered superior to general anesthesia, Black patients giving birth are 70% more likely than White patients giving birth to receive general anesthesia for cesarean delivery.^39^ Because untreated PDPH may result in chronic conditions and potentially serious complications, our study should be viewed as another call for action.

### Significance

Our findings have uncovered a gap in the quality of PDPH management provided to patients in racial and ethnic minority groups giving birth in New York State. It is incumbent on anesthesiologists to understand the factors associated with the disparities at the patient, clinician, and system level and to be a part of the solution.^40^

Standardizing care so that all patients who received neuraxial anesthesia or analgesia for childbirth are systematically evaluated by an anesthesia professional for the presence of headache prior to hospital discharge is critical. Obstetricians and nurses should receive training on the typical signs and symptoms of PDPH, and these signs and symptoms should be added to early warning criteria.^41^ Patients themselves should receive clear information in their primary language about their individual risk of headache, should be advised to report these symptoms to their clinicians, and should understand how to seek care. Although presumably similar disparities exist in other states, the lack of adequate identity and anesthesia-related outcomes data nationwide limits the ability to quantify disparities, investigate their causes, and institute corrective measures.^27^

### Limitations

Our findings should be interpreted in the context of the limitations of administrative data and *ICD* codes. First, we were unable to determine explanatory factors for the differences in EBP rates by race and ethnicity. We were unable to determine whether EBP was offered but declined, nor could we determine the possible role of implicit bias among health care professionals. Because the lowest EBP rates were among non-Hispanic Black patients and not Hispanic patients, it is unlikely that a language barrier was the reason for lower rates among the former group of patients. Second, our analysis was limited to delivery hospitalizations and did not account for PDPH diagnosed or EBP performed during a hospital readmission. Approximately 5% of PDPH cases are identified during a hospital readmission.^10^ Third, we used the interval between hospital admission and EBP as a proxy for the interval between the initial neuraxial procedure causing the PDPH and timing of the EBP. Although it may be inaccurate, it should not differentially affect White patients or patients in racial and ethnic minority groups. Fourth, we used the number of physician anesthesiologists at the hospital and county level as a proxy for the number at the individual hospital level because no current data system provides this information. Although this approach may be accurate for counties with only 1 hospital, it may not be accurate for counties with more than 1 hospital. Fifth, we had no information on the type of neuraxial procedure (spinal, epidural, or combined spinal epidural), the needle type (cutting or atraumatic), or the needle size that resulted in PDPH. To our knowledge, the accuracy of the variable anesthesia care, using individual medical records as the criterion standard, has never been studied. Sixth, our findings apply to New York State and cannot necessarily be generalized to other parts of the US because of the wide variation in the use of neuraxial procedures (labor analgesia rates or general anesthesia rates for cesarean delivery) across US states.^42^

## Conclusions

This cross-sectional study found that obstetric patients in racial and ethnic minority groups are less likely than White patients to receive EBP for PDPH during their delivery hospitalization, and, when they received EBP, they received it later than White patients. This failure to provide equitable obstetric anesthesia care must be addressed given the serious complications associated with PDPH.^10,11,43^

## Notes

Supplement. **eTable 1.** *ICD-9-CM* and *ICD-10-CM* Codes Used in This Study **eTable 2.** Imputation of Missing Values for the Multivariable Analysis of Risk Factors for Epidural Blood Patch Use **eFigure.** Flowchart of the Study **eTable 3.** Multivariable Analysis of Risk Factors for Use of Epidural Blood Patch for Postdural Puncture Headache After Neuraxial Analgesia or Anesthesia for Childbirth (New York State Hospitals, 1998-2016)
