# Supplementary material for: Racial and Ethnic Disparities in the Management of Postdural Puncture Headache With Epidural Blood Patch for Obstetric Patients in New York State
Source: JAMA Netw Open. 2022 Apr 21;5(4):e228520. doi: 10.1001/jamanetworkopen.2022.8520 (PMC9024387; doi:10.1001/jamanetworkopen.2022.8520)
Supplement: Supplement. — eTable 1. ICD-9-CM and ICD-10-CM Codes Used in This Study eTable 2. Imputation of Missing Values for the Multivariable Analysis of Risk Factors for Epidural Blood Patch Use eFigure. Flowchart of the Study eTable 3. Multivariable Analysis of Risk Factors for Use of Epidural Blood Patch for Postdural Puncture Headache After Neuraxial Analgesia or Anesthesia for Childbirth (New York State Hospitals, 1998-2016) [file jamanetwopen-e228520-s001.pdf]

Supplementary Online Content

Lee A, Guglielminotti J, Janvier AS, Li G, Landau R. Racial and ethnic disparities in the management of postdural puncture headache with epidural blood patch for obstetric patients in New York State. *JAMA Netw Open.* 2022;5(4):e228520. doi:10.1001/jamanetworkopen.2022.8520

eTable 1. *ICD-9-CM* and *ICD-10-CM* Codes Used in This Study
eTable 2. Imputation of Missing Values for the Multivariable Analysis of Risk Factors for Epidural Blood Patch Use
eFigure. Flowchart of the Study
eTable 3. Multivariable Analysis of Risk Factors for Use of Epidural Blood Patch for Postdural Puncture Headache After Neuraxial Analgesia or Anesthesia for Childbirth (New York State Hospitals, 1998-2016)

This supplementary material has been provided by the authors to give readers additional information about their work.

© 2022 Lee A et al. *JAMA Network Open*.

eTable 1. *ICD-9-CM* and *ICD-10-CM* Codes Used in This Study

	ICD-9-CM	***ICD-10-CM***
**Patient characteristics**		
Obesity	278.0,<br>649.1,<br>V85.3,<br>V85.4	E66,<br>O99.21,<br>Z68.3,<br>Z68.4
**Delivery mode**		
Cesarean delivery	74.0-74.2 (proc.),<br>74.4 (proc.),<br>74.9 (proc.)	O82,<br>10D00Z0 (proc.),<br>10D00Z1 (proc.),<br>10D00Z2 (proc.)
**Possible contraindications to neuraxial analgesia or anesthesia**		
Coagulation factor deficit,<br>Von Willebrand disease,<br>and thrombocytopenia	286,<br>287	D65-D69
Fever or infection during labor	659.2,<br>659.3	O75.2,<br>O75.3
Chorioamnionitis	658.4	O41.1

Abbreviations: proc.: procedure code.

© 2022 Lee A et al. *JAMA Network Open*.

eTable 2. Imputation of Missing Values for the Multivariable Analysis of Risk Factors for Epidural Blood Patch Use (package *mice* in R with 5 iterations and 5 datasets created)

**Variables with missing values**	**Number of missing values**
Teaching hospital	1153
Rural hospital	1153
Hospital volume of delivery	88
Hospital cesarean delivery rate	88
Hospital proportion of racial and ethnic minority parturients	178
Hospital proportion of safety net parturients	88
Hospital proportion of admission for delivery during a weekend	88
Hospital proportion of neuraxial analgesia or anesthesia for deliveries	88
Hospital coding intensity in deliveries	88
Hospital county number of obstetricians and gynecologists	1510
Hospital county number of physician anesthesiologists	1510
**Variables used to estimate missing values**	
**1. Outcome**	
Epidural blood patch	
**2. Patient characteristics**	
Age	
Health insurance	
Obesity	
Comorbidity index for obstetric patients	
**3. Delivery**	
Admission during a weekend	
Cesarean delivery	
**4. Contraindications to neuraxial techniques**	
Coagulation factor deficit, Von Willebrand disease, and thrombocytopenia	
Fever or infection during labor	
Chorioamnionitis	
**5. Other**	
Year of delivery	

© 2022 Lee A et al. *JAMA Network Open*.

eFigure. Flowchart of the Study
Abbreviations: EBP: epidural blood patch; ICD: international classification of diseases; PDPH: postdural puncture headache

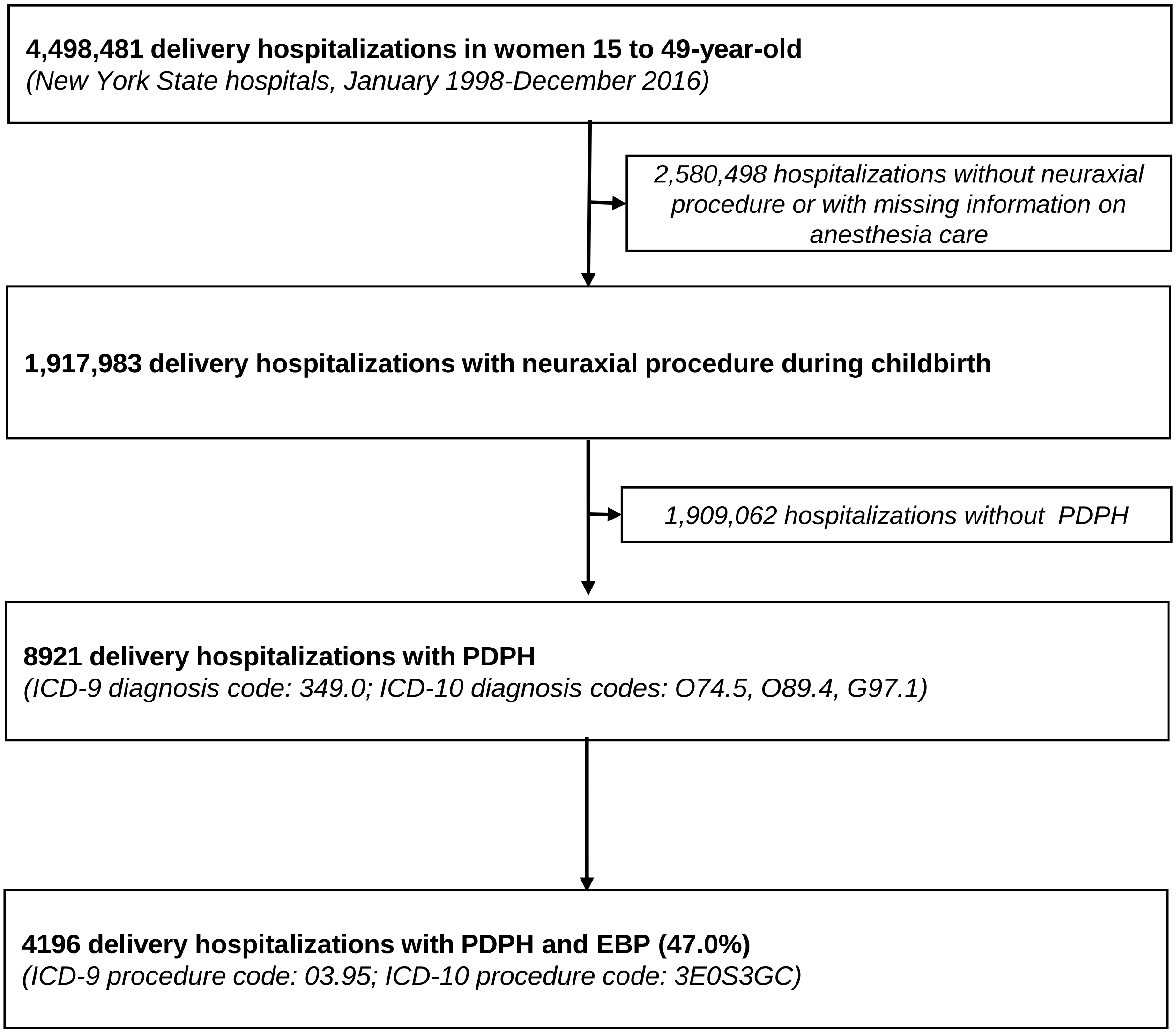

© 2022 Lee A et al. *JAMA Network Open*.

eTable 3. Multivariable Analysis of Risk Factors for Use of Epidural Blood Patch for Postdural Puncture Headache After Neuraxial Analgesia or Anesthesia for Childbirth (New York State Hospitals, 1998-2016)

	**Adjusted Odds Ratio (95% confidence interval)**
**Patient characteristics**	
Race and ethnicity	
White	1.000 (Reference)
Hispanic	1.106 (0.944, 1.296)
Black	***0.796 (0.671, 0.944)***
Other (**a**)	***0.846 (0.726, 0.986)***
Missing	1.313 (0.985, 1.750)
Age (per 1-year increase)	0.998 (0.989, 1.006)
Health insurance	
Medicaid or Medicare	1.000 (Reference)
Private	***1.192 (1.058, 1.344)***
Self-pay	0.973 (0.677, 1.398)
Other	***0.608 (0.425, 0.869)***
Obesity	0.879 (0.705, 1.096)
Comorbidity index for obstetric patients (per 1-unit increase)	0.994 (0.977, 1.012)
**Delivery**	
Admission for delivery during a weekend	1.136 (1.009, 1.278)
Cesarean delivery	***0.677 (0.607, 0.754)***
**Contraindications to neuraxial techniques**	
Coagulation factor deficit, Von Willebrand disease, and thrombocytopenia	0.809 (0.544, 1.203)
Fever or infection during labor	1.541 (0.959, 2.476)
Chorioamnionitis	0.888 (0.650, 1.213)
**Hospital characteristics**	
Teaching hospital	0.867 (0.739, 1.017)
Rural hospital	1.094 (0.829, 1.445)
Volume of delivery (per 100-birth increase)	1.000 (0.993, 1.006)
Cesarean delivery rate (per 1% increase)	1.011 (0.998, 1.025)
Proportion of racial and ethnic minority parturients (per 1% increase)	***0.994 (0.991, 0.998)***
Proportion of safety net parturients (per 1% increase)	***0.994 (0.989, 0.998)***
Proportion of admissions for delivery during a weekend (per 1% increase)	1.002 (0.972, 1.032)
Proportion of neuraxial analgesia or anesthesia for delivery (per 1% increase)	1.002 (1.000, 1.004)
Coding intensity in deliveries (per 1-unit increase)	0.980 (0.928, 1.035)
**Hospital county characteristics**	
Number of obstetricians and gynecologists (per 1 physician per 1000 birth increase)	***0.976 (0.960, 0.992)***
Number of physician anesthesiologists (per 1 physician per 1000 birth increase)	1.008 (0.995, 1.021)

© 2022 Lee A et al. *JAMA Network Open*.

eTable 3 (continued)

	**Adjusted Odds Ratio (95% confidence interval)**
**Year of delivery**	
1998	1.000 (Reference)
1999	0.786 (0.591, 1.045)
2000	0.882 (0.660, 1.179)
2001	0.837 (0.634, 1.105)
2002	0.774 (0.577, 1.039)
2003	0.759 (0.566, 1.019)
2004	0.937 (0.693, 1.267)
2005	0.777 (0.569, 1.062)
2006	0.862 (0.628, 1.183)
2007	0.774 (0.564, 1.063)
2008	0.851 (0.613, 1.181)
2009	***0.692 (0.497, 0.965)***
2010	0.733 (0.525, 1.024)
2011	0.776 (0.550, 1.094)
2012	0.994 (0.705, 1.400)
2013	0.951 (0.669, 1.351)
2014	1.084 (0.766, 1.535)
2015	0.930 (0.649, 1.333)
2016	0.417 (0.283, 0.614)

(**a**) Includes Asian and Pacific Islander, Native American, and Other race and ethnicity

© 2022 Lee A et al. *JAMA Network Open*.
